# Exogenous melatonin improved photosynthetic efficiency of photosystem II by reversible phosphorylation of thylakoid proteins in wheat under osmotic stress

**DOI:** 10.3389/fpls.2022.966181

**Published:** 2022-08-02

**Authors:** Shuai Lin, Xiao-Fang Song, Hao-Tian Mao, Shuang-Qing Li, Jie-Ying Gan, Ming Yuan, Zhong-Wei Zhang, Shu Yuan, Huai-Yu Zhang, Yan-Qiu Su, Yang-Er Chen

**Affiliations:** ^1^College of Life Sciences, Sichuan Agricultural University, Ya’an, China; ^2^College of Resources, Sichuan Agricultural University, Chengdu, China; ^3^College of Life Sciences, Sichuan Normal University, Chengdu, China

**Keywords:** wheat, melatonin, drought, PSII, protein phosphorylation

## Abstract

It has been well demonstrated that melatonin plays an important protective role in photosynthesis of plants under various environmental stresses, while the detailed mechanisms by which melatonin protects photosystem II (PSII) under environmental stress are still unclear. In the study, the effects of melatonin on photosynthetic efficiency, energy dissipation, PSII protein composition, and reversible phosphorylation of thylakoid proteins were investigated in wheat plants under osmotic stress. The results showed that osmotic stress significantly reduced pigment content, photochemical efficiency of PSII, oxygen-evolving activity, and dissipation of excess excitation energy, while 25 μM melatonin applications greatly alleviated their decline under osmotic stress. Western blot data of PSII proteins revealed that melatonin upregulated the levels of D1, Lhcb5, Lhcb6, PsbQ, and PsbS proteins in wheat exposed to osmotic stress. In addition, thylakoid membrane proteins were strongly phosphorylated in wheat under osmotic stress with or without melatonin. Furthermore, the results from PSII protein dephosphorylation showed that exogenous melatonin promoted the dephosphorylation of LCHII, CP43, and D1 under osmotic stress. Therefore, our findings suggest that melatonin can provide an effective protection for the photosynthetic apparatus by the regulation of PSII proteins and the reversible phosphorylation of thylakoid proteins under drought stress.

## Introduction

Drought is a major threat for plant growth and agricultural crop yields in the world and can affect many physiological and biochemical processes in plants. Water deficit stress has become a key constraint for agricultural development and production due to global warming in recent years, especially in arid and semi-arid regions (Avramova et al., 2015; Baghbani-Arani et al., 2017; Li et al., 2019). During water shortage, plants experience a number of metabolic changes, including gene expression, protein synthesis, pigment contents, antioxidant system, accumulation of reactive oxygen species (ROS), biomass, and yield (Chen et al., 2016a, 2017a; Song et al., 2020; Zhao et al., 2020). Drought can drastically affect the performance of the photosynthetic apparatus directly or indirectly (Xing and Wu, 2012; Hazrati et al., 2016). The changes in photosynthetic performance under drought stress mainly contain reduction of the intercellular CO_2_ concentration, inactivation of PSI and PSII reaction center, decrease in photosynthetic pigment content, decline in the amount of PSII proteins, and disassembly of PSII-LHCII supercomplexes (Chen et al., 2016a, 2017a; Baghbani-Arani et al., 2017; Song et al., 2020).

Photosystem II, a large multi-subunit pigment-protein complex embedded in the thylakoid membranes of photosynthetic organisms, mainly contains reaction centers (RCs), the chlorophyll a/b light-harvesting complex (LHCII), and the oxygen-evolving complex (OEC; Allahverdiyeva et al., 2013). PSII of vascular plants is very sensitive to different stresses and usually suffers from different damage under environmental stresses. Many researches have indicated that RCs are the main component damaged by different environmental stresses (Sperdouli and Moustakas, 2012; Chen et al., 2016a, 2017a). As the key member of PSII RCs, D1 protein has been shown to be the most sensitive target under environmental stresses (Li et al., 2016; Chen et al., 2017b). In addition, drought stress may inhibit the electron transfer in PSII, thus results in the generation of ROS in the thylakoid membrane, and finally reduces the efficiency of PSII (Baghbani-Arani et al., 2017; Chen et al., 2017a,b). It has been well known that many thylakoid membrane proteins including D1, D2, CP43, LHCII, and PsbH in photosynthetic apparatus can undergo rapid phosphorylation and dephosphorylation in response to environmental changes. Our previous study showed that two wheat cultivars with different drought resistance presented different patterns in the reversible phosphorylation of PSII proteins under osmotic stress (Chen et al., 2017a). Therefore, how to improve the photosynthetic efficiency of PSII has been an important area of research under drought stress.

N-acetyl-5-methoxytryptamine (Melatonin, MT), as a new type of hormone-like substance, is a small and natural indoleamine compound that was first isolated from pineal gland of cows (Lerner et al., 1958). In 1995, melatonin was identified in plants (Dubbels et al., 1995; Hattori et al., 1995). In vascular plants, melatonin plays the vital role in regulating seed germination, improving root development, and simulating plant growth (Arnao and Hernandez-Ruiz, 2015). Furthermore, MT is also involved in the regulation of signaling molecule that takes part in the response of plants to abiotic and biological stresses (Shi et al., 2015; Arora and Bhatla, 2017). In addition, it has been demonstrated that MT can improve plant tolerance to abiotic stresses, such as drought stress, cold stress, salinity stress and heavy metal stress (Zhang et al., 2014, 2021; Chen et al., 2018a; Huang et al., 2019; Shah et al., 2021), and biotic stresses (Yin et al., 2013; Moustafa-Farag et al., 2020). Many evidences have demonstrated that melatonin improves photosynthetic capacity and provides the effective protective role in photosynthesis *via* inhibiting the expression of chlorophyll degradation-associated genes or upregulating the expression of chlorophyll-synthesis-related genes (Yang et al., 2022), increasing the expression levels of photosynthesis-related proteins (Wang et al., 2022), maintaining the fluidity of thylakoid membrane (Khan et al., 2019), facilitating the recovery of D1 protein synthesis and photosynthetic electron transport chain (Zhou et al., 2016), and participating in the ABA signal pathway (Wang et al., 2021) or calcium signal transduction (Wang et al., 2022) under stress. However, the detailed protective mechanism of melatonin in PSII against drought stress is still very limited to be clarify, specially for the reversible phosphorylation of thylakoid membrane proteins.

Wheat (*Triticum aestivum* L.) is one of the three main food crops cultivated worldwide (Chen et al., 2018b). However, drought can severely affect the physiological and biochemical processes of wheat plants (Chen et al., 2017a), and thus decreases wheat productivity and quality (Daryanto et al., 2016; Zhao et al., 2020). Although it has been demonstrated that melatonin is associated with the protection for photosynthesis in response to environmental stresses (Chen et al., 2018a; Zhang et al., 2021), the detailed protective mechanism of exogenous melatonin on PSII has not been explored thoroughly in wheat. Therefore, we investigated the protective role of different concentrations of melatonin in wheat PSII under osmotic stress. In addition, the potential mechanism of melatonin in protecting photosynthetic apparatus was also proposed. Our study provides new insights into the improvement of stress resistance and photoprotection of PSII by melatonin in plants.

## Materials and methods

### Plant materials and treatments

Wheat (*Triticvum aestivm* L. Chuannong 19 cultivar., CN19) seeds were surface-sterilized using 1% sodium hypochlorite (NaClO) solution for 15 min and then germinated on petri dishes for 48 h in the dark at 25°C. The germinated seeds were planted in quartz sand with 1/2 strength Hoagland’s solution and transferred in the growth chamber with a 12/12 h (25/22°C) day/night, 75% relative humidity, and 250 μmol m^−2^ s^−1^ photo flux density. At the third-leaf-stage, wheat seedlings were used for different treatments. Osmotic stress was performed for 72 h as described by Chen et al. (2017a). All seedlings were randomly divided into eight groups: (1) 1/2 Hoagland medium; (2) 1/2 Hoagland medium + 20% (w/v) PEG-6000; (3) 1/2 Hoagland medium + 5 μM melatonin; (4) 1/2 Hoagland medium + 25 μM melatonin; (5) 1/2 Hoagland medium + 100 μM melatonin; (6) 1/2 Hoagland medium + 5 μM melatonin + 20% PEG-6000; (7) 1/2 Hoagland medium + 25 μM melatonin + 20% PEG-6000; (8) 1/2 Hoagland medium + 100 μM melatonin + 20% PEG-6000. After 72 h of treatments, chlorophyll fluorescence of wheat leaves was determined and thylakoid proteins were extracted according to our previous methods (Chen et al., 2016b, 2017b, 2019).

### Measurements of pigment, relative water content, and leaf absorption

Chlorophyll were extracted from fresh wheat leaves (0.5 g) using acetone (80%, v/v) and determined by a UV spectrophotometer (Hitachi-U2000, Hitachi, Ltd., Tokyo, Japan) as following our previous method (Chen et al., 2018a). Relative water content (RWC) of the leaves was measured according to the method described by Chen et al. (2017a). Leaf absorption was determined using BioMate 3S UV–Vis spectrophotometer with a mounted integrating sphere based on the previous method (Bielczynski et al., 2020).

### Chlorophyll fluorescence, NPQ kinetic, and state transition measurements

Chlorophyll (Chl) fluorescence of PSII was analyzed at room temperature using a modulated imaging fluorometer (the Imaging PAM M-Series Chlorophyll Fluorescence System, Heinz-Walz Instruments, Effeltrich, Germany) according to our previous methods (Chen et al., 2017b, 2018b). Before the measurements of Chl fluorescence, wheat samples were put in the dark for 1 h. The saturation pulse intensity of 8,000 μmol m^−2^ s^−1^ and the actinic light intensity of 150 μmol m^−2^ s^−1^ are given. The maximum efficiency of PSII photochemistry (*F*v/*F*m), quantum yield of non-regulated energy dissipation [Y(NO)], non-photochemical quenching coefficient (qN), quantum yield of regulated energy dissipation [Y(NPQ)], effective PS II quantum yield [Y(II)], and coefficient of photochemical quenching (qL and qP) were imaged and calculated as following previous method (Maxwell and Johnson, 2000).

Dual PAM-100 fluorometer (Heinz-Walz Instruments, Effeltrich, Germany) was used to measure NPQ kinetic and state transition in whole plants as the previous method described (Pietrzykowska et al., 2014). Wheat plants were adapted in dark for 1 h prior to measurements. The NPQ kinetics was obtained based on the *F*m value measured from an untreated plant. At the end of each state, the level of *F*m in State I (*F*m′) and State II (*F*m″) was recorded using the application of the saturating light pulse.

### OJIP transients, gas exchange, and oxygen-evolving activity

Determination of the fast phase of Chl *a* fluorescence induction (FI) was performed using a dual PAM-100 fluorometer (Heinz-Walz Instruments, Effeltrich, Germany) following the method described by Tang et al. (2020). In the measurement of OJIP transients, the plants were adapted for at least 30 min in the dark. Then, the adaxial surface of the leaves was exposed to saturated pulse intensity (5,000 μmol photons m^−2^ s^−1^) for 0.5 s. The OJIP steps referred to the minimal fluorescence intensity when all PSII reaction centers (RCs) were open (O step), the intensity at 2 ms (J step), the intensity at 30 ms (I step), and the maximal intensity when all PSII RCs were closed (P step). OJIP curves were standardized by O-P to make V_O-P_ curves following the method of Tang et al. (2020).

The GSF-3000 photosynthesis system (Heinz-Walz Instruments, Effeltrich, Germany) was used to determine intercellular CO_2_ concentration (*C_i_*), stomatal conductance (*G_s_*), net photosynthetic rate (*P_n_*), and transpiration rate (*T_r_*) of wheat leaves in different treatments. To measure CO_2_ assimilation rate, 360 μmol mol^−1^ CO_2_ concentration, 60–80% relative humidity, and illumination of 1,000 μmol photons m^−2^ s^−1^ at 25°C were given.

Oxygen-evolving activity of thylakoid membranes obtained from the leaves was determined with a Clark-type electrode (Hansatech, Norfolk, United Kingdom) in a buffer containing the artificial electron acceptor phenyl-*p*-benzoquinone (PpBQ, 0.25 mM), 25 mM Hepes (pH 7.6), 0.2 M sucrose, 10 mM NaCl, and 5 mM CaCl_2_ at 20°C (Chen et al., 2016b).

### Isolation of thylakoid membrane proteins and immunoblotting analysis

Isolation of thylakoid proteins from wheat leaves was performed at 4°C under dim light based on our previous method (Chen et al., 2016b). The chlorophyll concentration of thylakoid membranes was measured following the previous method (Porra et al., 1989). Then, SDS-PAGE (6% acrylamide stacking gel + 15% separation gel + 6 M urea) was used to separate thylakoid membrane proteins (Laemmli, 1970). The separated proteins was transferred to polyvinylidene fluoride membrane (Immobilone, Millipore, Darmstadt, Germany), and subsequently were immunodetected with specific antibodies against D1, CP43, CP47, Lhcb4, Lhcb5, Lhcb6, PsbO, PsbP, PsbQ, PsbS, and P-Lhcb2 (Agrisera, Umea, Sweden). For protein phosphorylation assays, all buffers were supplemented with 10 mM NaF and the transferred thylakoid proteins were detected with the anti-phosphothreonine antibody (Cell Signaling, Ipswich, MA, United States). To detect the immunoblotting signals, the horseradish peroxidase-conjugated secondary antibody (Agrisera, Umea, Sweden) and a chemiluminescent detection system (ECL, GE Healthcare, Buckinghamshire, United Kingdom) was adopted. Quantification of signal amplitude was obtained using Quantity One software (Bio-Rad Co., Hercules, CA, United States).

For dephosphorylation analyses of PSII proteins, wheat seedling were exposed to 1,000 μmol photons m^−2^ s^−1^ or 80 μmol photons m^−2^ s^−1^ PPFD for 60 min to induce the phosphorylation of PSII reaction center proteins or maximum LHCII phosphorylation (Chen et al., 2017a), respectively. After these treatments, the samples were kept in the dark for 120 min to induce gradual dephosphorylation of thylakoid proteins in the greenhouse. Then, thylakoid membranes of wheat samples frozen in liquid nitrogen were extracted during the time course of incubation, and stored at −80°C according to the method of Chen et al. (2016b).

### Blue native PAGE and 2D electrophoresis

Blue native-polyacrylamide gel electrophoresis (BN-PAGE) analysis was performed according to our previous method (Chen et al., 2016b). Thylakoid membranes (20 μg of Chl) were solubilized using 1% (w/v) *n*-dodecyl-*β*-D-maltoside (Sigma Chemical Co. St. Louis, MO, United States) in the dark for 10 min on ice. BN-PAGE was performed with a gradient of 5–12.5% acrylamide in the separation gel and a gradual increase in the voltage (75–200 V) for 3–4 h at 4°C. For the second-dimensional separation, the BN-PAGE strips were cut and then incubated in Laemmli (1970) buffer containing 2-mercaptoethanol (5%, v/v) for 1 h at room temperature prior to SDS-PAGE including 15% acrylamide and 6 M urea. After SDS-PAGE, the proteins were stained by Coomassie Brilliant Blue R.

### Statistical analysis

The data were analyzed using the SPSS Statistics 19.0 software (IBM, Chicago, IL, United States), followed by Duncan’s multiple range tests, and were presented as the mean ± SD of three independent replicates. Different letters indicated to be statistically significant at the 0.05 level among treatments.

## Results

### Effects of exogenous melatonin on phenotype, RWC, and pigment contents under osmotic stress

To test the protective roles of melatonin in plant growth, the phenotype of CN19 was observed in the presence or absence of four different concentrations of melatonin (0, 5, 25, and 100 μM) under osmotic stress for 3 days (Supplementary Figure S1A). Compared with the control, the melatonin-treated seedlings showed no obvious difference in the phenotype under non-stress condition, while osmotic stress resulted in a more obvious wilting. In contrast, 5 and 25 μM melatonin application greatly alleviated the wilting of wheat seedlings under osmotic stress. It was noted that 100 μM melatonin treatment did not show the similar results under stress condition. In addition, RWC significantly decreased in CN19 under osmotic stress relative to the control (Supplementary Figure S1B). Under osmotic stress for 72 h, melatonin treatments markedly alleviated the decline in RWC.

**Figure 1:**
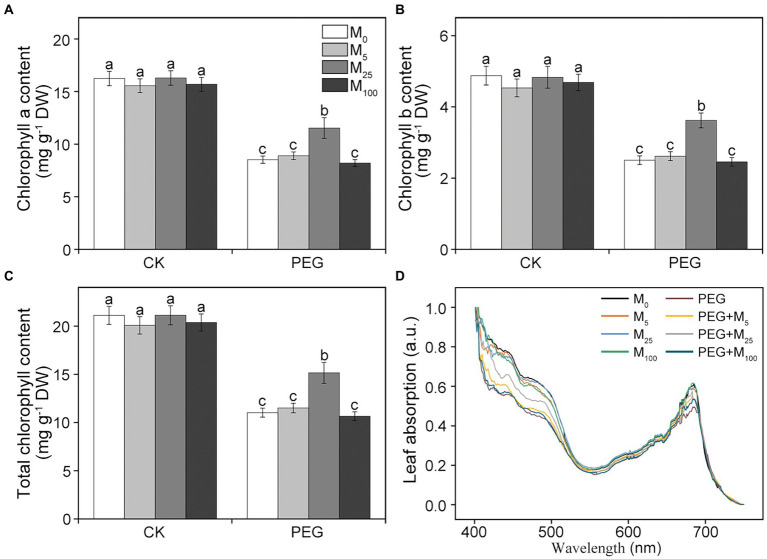
Effects of melatonin on chlorophyll *a* **(A)**, chlorophyll *b* **(B)**, total chlorophyll content **(C)**, and leaf absorption **(D)** in wheat seedlings under osmotic stress. Bars represent SDs from three independent biological replicates (*n* = 3). Different letters indicate significant differences (*p* < 0.05) according to Duncan’s multiplication range test. CK, non-stressed wheat plants; PEG, osmotic stress for 3 days; and M_0_–M_100_ show 0, 5, 25, and 100 μM melatonin, respectively.

Chlorophyll (Chl) and leaf absorption were further determined in this experiment. Compared to the control, osmotic stress resulted in the significant decline in Chl *a*, Chl *b*, and total Chl content (Figures 1A–C). When osmotic stress was applied, 25 μM melatonin treatment markedly improved the level of chlorophyll. Similarly, leaf absorption significantly decreased at 400–500 and 680 nm under osmotic stress relative to the control (Figure 1D). However, 5 and 25 μM melatonin obviously alleviated the decline in leaf absorption under osmotic stress, in agreement with a decreased Chl content. These results indicated that exogenous melatonin application could protect photosynthetic pigments of wheat plants under osmotic stress.

### Melatonin improved photosynthetic efficiency under osmotic stress

To explore whether melatonin could improve the photosynthetic capacity under environmental stresses, gas exchange parameters and chlorophyll fluorescence were examined in wheat seedlings treated with PEG-6000 and melatonin, respectively. As depicted in Supplementary Figure S2, net photosynthetic rate (*P_n_*), transpiration rate (*T_r_*), and stomatal conductance (*G_s_*) were greatly reduced, whereas intercellular CO_2_ concentration (*C_i_*) increased in non-melatonin-treated CN19 seedlings under osmotic stress. Additionally, melatonin treatments significantly relieved the decline in *P_n_*, *T_r_*, and *G_s_*, and the increase in *C_i_* induced by osmotic stress, especially for 25 μM melatonin.

**Figure 2:**
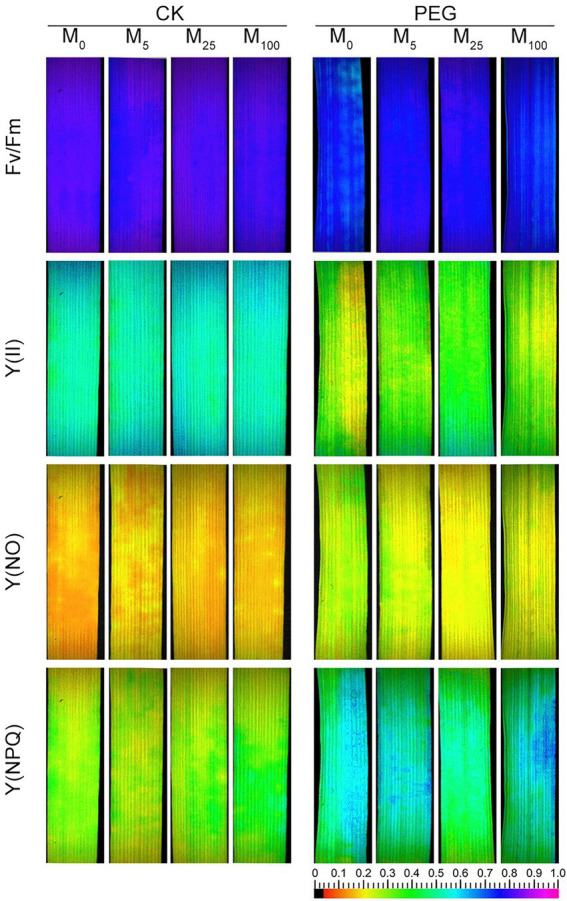
Effects of melatonin on chlorophyll fluorescence parameters [*F*v/*F*m, maximum efficiency of PSII photochemistry; Y(II), effective PSII quantum yield; Y(NO), quantum yield of non-regulated energy dissipation; and Y(NPQ), quantum yield of regulated energy dissipation] in wheat seedlings under osmotic stress. CK, non-stressed wheat plants; PEG, osmotic stress for 3 days; and M_0_–M_100_ show 0, 5, 25, and 100 μM melatonin, respectively.

**Figure 3:**
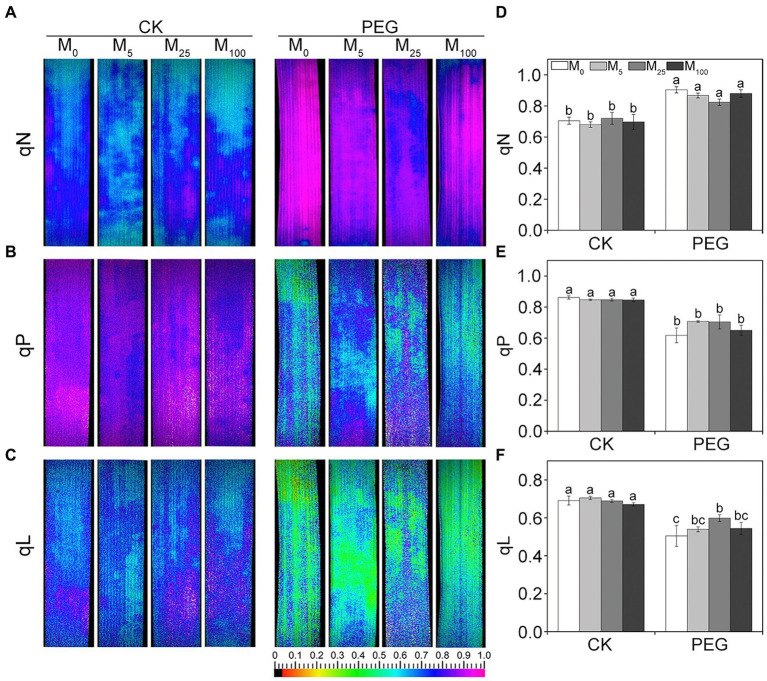
Effects of melatonin on quenching coefficient in wheat seedlings under osmotic stress. qN **(A)**, non-photochemical quenching coefficient. qP **(B)** and qL **(C)** are photochemical quenching coefficient based on the “puddle mode” and “lake model,” respectively. **(D–F)** are quantitative values of qN, qP, and qL, respectively. Bars represent SDs from three independent biological replicates (*n* = 3). Different letters indicate significant differences (*p* < 0.05) according to Duncan’s multiplication range test. CK, non-stressed wheat plants; PEG, osmotic stress for 3 days; and M_0_–M_100_ show 0, 5, 25, and 100 μM melatonin, respectively.

Considering the changes in gas exchange parameters, PSII photochemistry in wheat plants exposed to osmotic stress with or without melatonin was further determined. Under non-stress conditions, no obvious differences was seen in Fv/Fm, Y(II), Y(NO), and Y(NPQ) between melatonin-treated and non-melatonin-treated seedlings (Figure 2; Supplementary Figure S3). However, osmotic stress resulted in a significant decrease in Fv/Fm and Y(II), and a remarkable increase in Y(NO) and Y(NPQ). Under osmotic stress, 25 μM melatonin-treated CN19 seedlings displayed lower level of Y(NO) and Y(NPQ), and higher value of Fv/Fm and Y(II) relative to non-melatonin-treated CN19. These changes in Y(II), Y(NO), and Y(NPQ) were further verified by their light response curves (Supplementary Figures S4A–C). Furthermore, non-photochemical quenching coefficient (qN) and coefficient of photochemical quenching (qL and qP) significantly increased and decreased in CN19 exposed to osmotic stress compared with the control (Figure 3; Supplementary Figures S4D–F), respectively. However, melatonin treatments could effectively alleviate the increase or decline under osmotic stress, especially for 25 μM melatonin.

**Figure 4:**
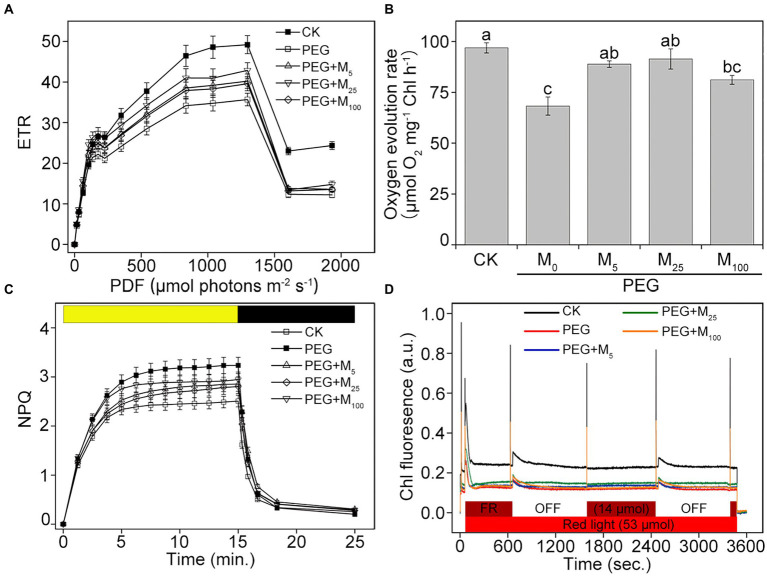
Effects of melatonin on ETR **(A)**, oxygen evolution **(B)**, NPQ **(C)**, and state transitions **(D)** in wheat seedlings under osmotic stress. ETR, electron transport rate. NPQ, non-photochemical quenching. Bars represent SDs from three independent biological replicates (*n* = 3). Different letters indicate significant differences (*p* < 0.05) according to Duncan’s multiplication range test. CK, non-stressed wheat plants; PEG, osmotic stress for 3 days; and M_0_–M_100_ show 0, 5, 25, and 100 μM melatonin, respectively.

In addition, the steady-state levels of ETR were significantly reduced in osmotic-stressed plants under medium and high actinic light intensities compared with the control (Figure 4A). However, melatonin treatments could lower the decline in ETR of CN19 seedlings under osmotic stress. Additionally, the rate of steady-state O_2_ evolution from isolated thylakoid membranes were investigated under osmotic stress (Figure 4B). Compared to the control, the capacity of O_2_ evolution significantly decreased in CN19 exposed to PEG-6000 for 3 days, whereas melatonin application greatly improved oxygen-evolving activity of thylakoid membranes under osmotic stress. Thus, these results indicated that exogenous melatonin played an important role in alleviating the damage to PSII and increasing the efficiency of electron transfer under environmental stresses.

### Melatonin increased dissipation of excess light energy under osmotic stress

To investigate the role of melatonin in energy dissipation under osmotic stress, two important photoprotection processes including NPQ and state transition were measured in wheat exposed to PEG-6000. Compared with the control, the inductions of NPQ in osmotic-stressed plants were faster and reached higher amplitude in the presence or absence of melatonin (Figure 4C). When osmotic stress was applied, melatonin treatments decreased the induction of NPQ during the periods of high-light illumination, specially for 25 μM melatonin, suggesting that melatonin could help to dissipate the excess energy under osmotic stress. Dark recovery was slower in PEG-6000-treated plants compared with the control plants, while melatonin application did not obviously promote dark recovery under osmotic stress. In addition, the obvious changes in state transitions was showed in Figure 4D. Compared to the control, PEG-6000 treatments in the presence or absence of melatonin resulted in the fast decrease in the capacity of the state I to state II transition in CN19. The final amplitudes of the state transitions in all stressed-plants were lower than those in the control. Under osmotic stress, 25 μM melatonin displayed a better kinetics of state transitions compared with other treatments. Therefore, the results indicated that exogenous melatonin could protect PSII through regulating energy dissipation under osmotic stress.

### Effects of melatonin on OJIP curves under osmotic stress

**Figure 5:**
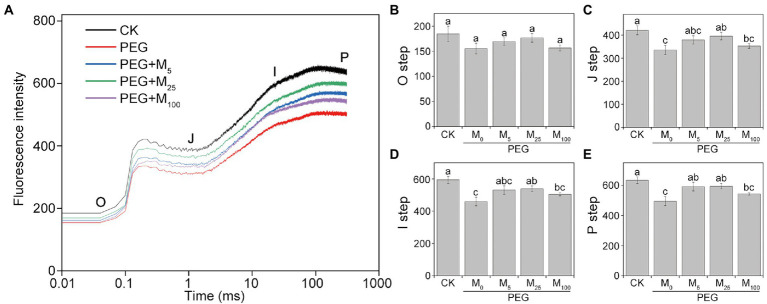
Effects of melatonin on chlorophyll a fluorescence OJIP transient curves **(A)** and the O-P steps **(B–E)** in wheat seedlings under osmotic stress. Each step shown in the figure indicate the minimal fluorescence intensity when all photosystem II (PSII) reaction centers (RCs) are open (the O step), the intensity at 2 ms (the J step), the intensity at 30 ms (the I step), and the maximal intensity when all PSII RCs are closed (the P step). Bars represent SDs from three independent biological replicates (*n* = 3). Different letters indicate significant differences (*p* < 0.05) according to Duncan’s multiplication range test. CK, non-stressed wheat plants; PEG, osmotic stress for 3 days; and M_0_–M_100_ show 0, 5, 25, and 100 μM melatonin, respectively.

As depicted in Figure 5, PEG-6000 treatments significantly altered the OJIP curve of CN19 leaves. In OJIP curves, each step exhibited a similar response to osmotic stress. Compared to the control, osmotic stress markedly reduced the relative fluorescence intensity of the J, I, and P step. However, melatonin treatment significantly alleviated the decline of point J, I, and P in wheat leaves under osmotic stress. Furthermore, OJIP curves of wheat leaves in different treatments were standardized by *V*_O-P_ (Supplementary Figure S5). The results showed that the relative variable fluorescence *V*_J_ at 2 ms of the *V*_O-P_ curve decreased significantly under osmotic stress, but *V*_J_ of melatonin-treated wheat was higher than that of non-melatonin-treated wheat.

### Effects of melatonin on PSII proteins and thylakoid complexes under osmotic stress

**Figure 6:**
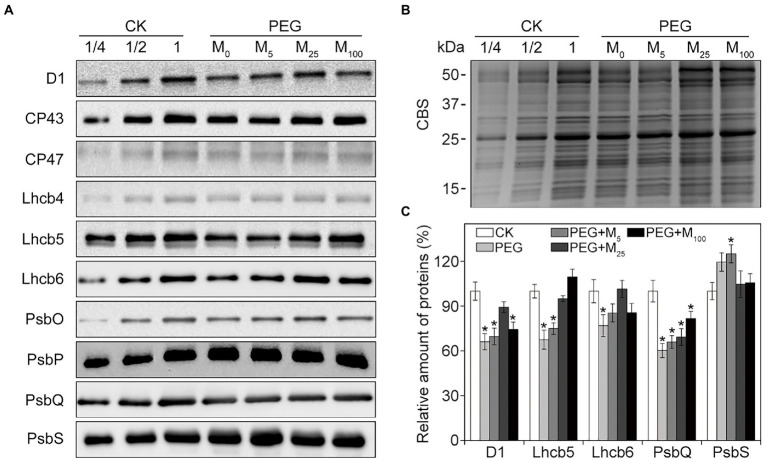
Immunoblot analyses of PSII proteins in wheat seedlings with different melatonin concentrations under osmotic stress. **(A)** Immunoblotting of thylakoid membrane proteins was performed using specific antibodies against D1, CP43, CP47, Lhcb4, Lhcb5, Lhcb6, PsbO, PsbP, PsbQ, and PsbS. **(B)** SDS-PAGE stained with Coomassie blue staining (CBS) are used as the control. **(C)** Quantification of the immunoblots indicates the relative abundances of the proteins relative to the amount of control (100%). Values are the averages of three replicates ±SD. Asterisks indicate statistically significant differences at *p* < 0.05 level (Duncan’s multiple range test). CK, non-stressed wheat plants; PEG, osmotic stress for 3 days; and M_0_–M_100_ show 0, 5, 25, and 100 μM melatonin, respectively.

To explore the detailed protective roles of melatonin in PSII, thylakoid protein composition was analyzed by immunoblotting under osmotic stress with or without melatonin application. As reported in Figure 6, osmotic stress led to the significant decline in the amount of D1, Lhcb5, Lhcb6, and PsbQ, and an increase in PsbS relative to the control. Conversely, melatonin application alleviated the negative influences of osmotic stress on these proteins, especially for 25 μM melatonin, suggesting that exogenous melatonin could inhibit the degradation of some PSII proteins under osmotic stress. Correspondingly, the organization of thylakoid membrane protein complexes was analyzed by BN-PAGE in wheat plants exposed to osmotic stress (Supplementary Figure S6). Relative to the control plants, thylakoid membrane protein complexes displayed no observed difference between melatonin-treated and non-treated seedlings under osmotic stress. The results were further identified by the data obtained from the second dimension gel (Supplementary Figure S7).

### Melatonin facilitated reversible phosphorylation of PSII proteins under osmotic stress

**Figure 7:**
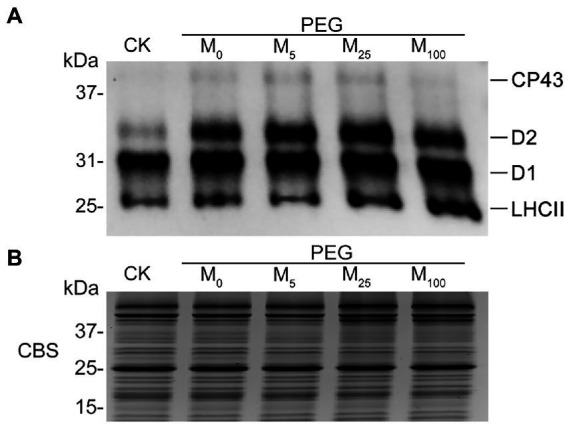
Phosphorylation of thylakoid proteins in wheat plants with different melatonin concentrations under osmotic stress. **(A)** Immunoblotting of PSII proteins was carried out using an anti-phosphothreonine antibody. Loading was based on an equal amount of chlorophyll (1 μg of total chlorophyll). **(B)** The results from Coomassie blue staining (CBS) of SDS-PAGE were given in the bottom panel. CK, non-stressed wheat plants; PEG, osmotic stress for 3 days; and M_0_–M_100_ show 0, 5, 25, and 100 μM melatonin, respectively.

**Figure 8:**
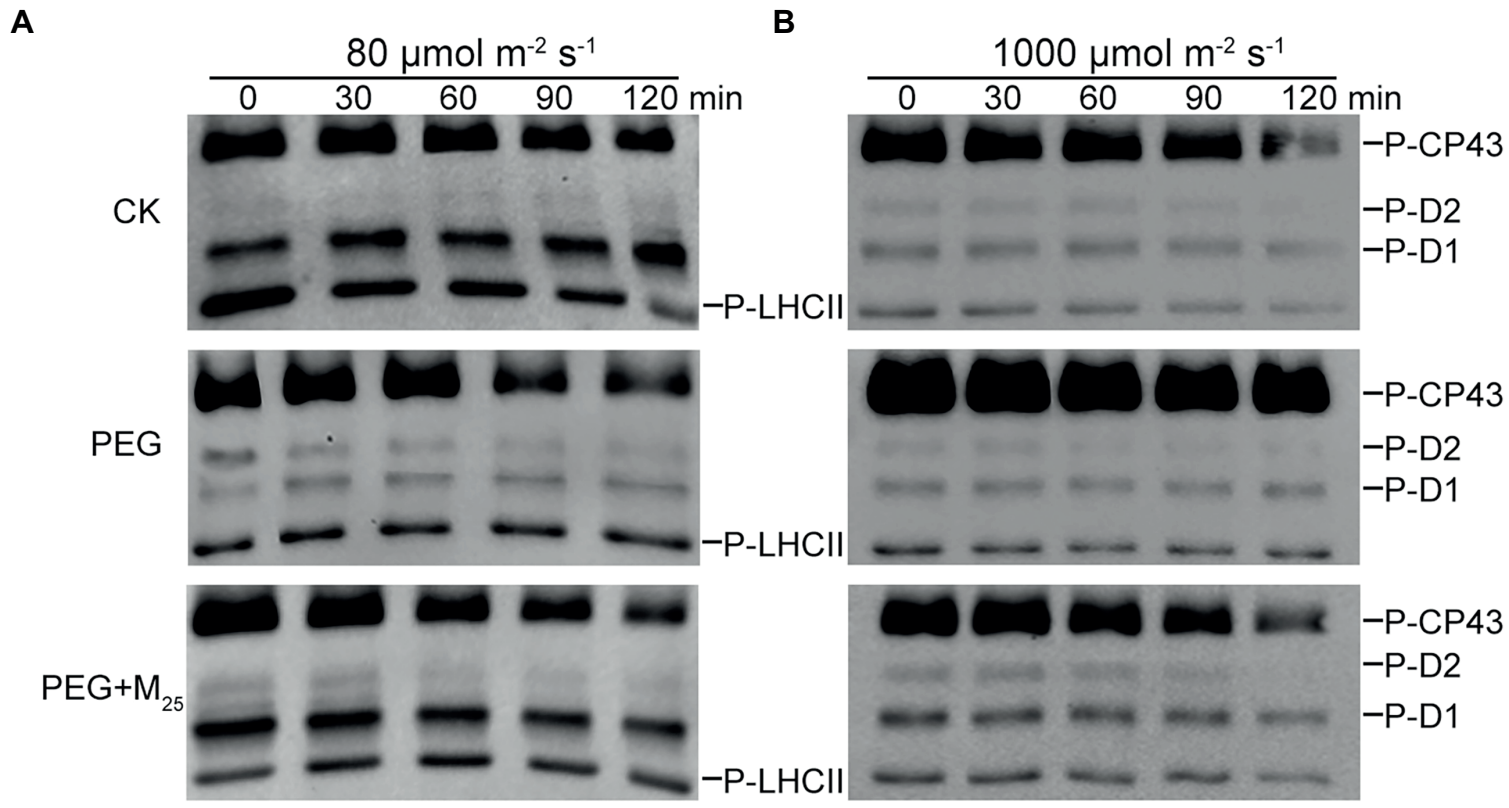
Dephosphorylation of PSII proteins from wheat seedlings with 25 μM melatonin *in vivo* under osmotic stress. Wheat seedlings were exposed to 80 μmol photons m^−2^ s^−1^ to induce maximal LHCII phosphorylation **(A)** or 1,000 μmol photons m^−2^ s^−1^ to phosphorylate PSII reaction center proteins **(B)** for 60 min at 25°C, and subsequently transferred to darkness at 25°C. Dephosphorylation was terminated at the indicated time points by freezing the leaves in liquid nitrogen. Thylakoid membranes were isolated and then the extent of protein phosphorylation was measured using a phosphothreonine antibody. CK, non-stressed wheat plants; PEG, osmotic stress for 3 days; and M_25_, 25 μM melatonin.

To test the regulatory role of melatonin in the reversible phosphorylation pattern of PSII proteins under osmotic stress, the phosphorylation and dephosphorylation of certain PSII subunits were determined in wheat plants exposed to different treatments, respectively. Compared with the control, osmotic stress induced the strong phosphorylation of CP43, D1, D2, and LHCII in the presence or absence of melatonin (Figure 7; Supplementary Figure S8). Under osmotic stress, melatonin treatment resulted in the different phosphorylation of four proteins. However, 25 μM melatonin led to the obvious accumulation of phosphorylated-D2 (P-D2), P-D1, and P-LHCII under osmotic stress. In addition, specific dephosphorylation of PSII proteins induced by osmotic stress *in vivo* was analyzed using the phosphothreonine antibody in wheat leaves. Under non-stress condition, LHCII rapidly dephosphorylated in the dark, especially at 120 min (Figure 8A; Supplementary Figure S9). In contrast, LHCII showed a slow dephosphorylation under osmotic stress without melatonin. However, 25 μM melatonin application resulted in a faster dephosphorylation under osmotic stress compared to the single PEG-6000 treatment (Supplementary Figure S9). The results of LHCII dephosphorylation were further identified by the level of Lhcb2 phosphorylation (Supplementary Figure S10), which is involved in the formation of the PSI-LHCII supercomplexes (Crepin and Caffarri, 2015). Furthermore, CP43, D1, and D2 presented the similar changes in dephosphorylation pattern with LHCII under osmotic stress with or without 25 μM melatonin compared to the control, specially at 120 min (Figure 8B). These results indicated that exogenous melatonin promoted the dephosphorylation of PSII proteins and thus accelerated protein turnover under osmotic stress.

## Discussion

Many studies have indicated that drought stress is one of the most key environmental stresses and severely influences plant growth and yield (Marcińska et al., 2013; Chen et al., 2016a). Our previous reports have demonstrated that many environmental stresses including osmotic stress could damage not only antioxidative system but also reduce photosynthetic capacity of PSII in different plants (Chen et al., 2016a, 2017a, 2018a; Huang et al., 2019). Melatonin, a new plant growth regulator, has been revealed to be related with the resistance to multiple abiotic stresses including salt stress, heavy metal stress, chilling, and drought stress (Zhang et al., 2015; Li et al., 2016; Chen et al., 2018a; Huang et al., 2019; Zhang et al., 2021). In addition, melatonin can trigger many genes and enzymes associated with nitrogen and carbon metabolism, and thus improved photosynthesis to promote the growth and development of plants under environmental stresses (Yan et al., 2021; Zhang et al., 2021). In the present experiment, the detailed protective roles of exogenous melatonin in PSII were investigated in wheat under osmotic stress.

Some previous studies have showed that melatonin could improve plant growth and alleviate the decline in photosynthetic pigments under environmental stresses (Chen et al., 2018a; Huang et al., 2019). In accordance with these findings, our results showed that melatonin application help to alleviate the stress phenotype and increase pigment content in wheat under osmotic stress. Many evidences demonstrated that melatonin mainly prevented chlorophyll degradation by downregulating the expression of chlorophyll degradation-associated genes or inhibited enzyme activities of chlorophyll catabolism (Jahan et al., 2020; Wu et al., 2021). Therefore, the increase in chlorophyll content was probably because that melatonin alleviated chlorophyll breakdown under osmotic stress. A previous study showed that melatonin with 100 μM played significant improvement in photosynthetic pigments under salt stress in maize (Chen et al., 2018a). However, the present experimental results indicated that 25 μM melatonin had an obvious impact on pigments and leaf absorption in wheat exposed to osmotic stress.

It has been known that stomatal closure could result in the decrease in photosynthetic capacity under environmental stresses (Farooq et al., 2009; Huang et al., 2019). However, a recent research implied that melatonin was probably involved in stomatal behavior by ABA signaling indirectly or directly participating in stomatal movement through the phytomelatonin receptor CAND2/PMTR1 (Yang et al., 2022). In the present study, osmotic stress greatly reduced *P*n, *T*r, and *G*s and increased *C*i, while these decline or improvement were partly recovered by the application of exogenous melatonin with 25 μM. These changes in gas exchange were probably because melatonin can alter the stomatal open state under osmotic stress (Huang et al., 2019). Similar findings were found in tomatoes exposed to drought stress (Liu et al., 2015).

Chlorophyll fluorescence has been an important and sensitive indicator for investigating plant photosynthetic characteristics under different environmental cues (Chen et al., 2017a, 2018b). Many studies have indicated that severe treatments may result in the decline in PSII photochemical efficiency and even the inactivation of PSII reaction center (Chen et al., 2018b; Huang et al., 2019; Zhang et al., 2021). In accordance with these findings, Fv/Fm, Y(II), qP, and qL markedly declined, while Y(NO), Y(NPQ), and qN significantly increased under osmotic stress. These changes suggested that osmotic stress caused serious damage to photosynthetic capacity of PSII in wheat. The low Y(II) reflects a low quantum yield of PSII (Chen et al., 2018b). Therefore, high value of Y(II) in the presence of melatonin probably suggested that exogenous melatonin promoted the linear electron flow under osmotic stress. Y(NO) and Y(NPQ) describe the quantum yield of non-regulated and regulated energy dissipation in PSII, respectively. Thus, low values of Y(NO) and Y(NPQ) reflected the there was no need for photoprotection in the presence of melatonin under osmotic stress (Chen et al., 2018b; Moustakas et al., 2022). qP and qL are the measurements of the fraction of open PSII reaction centers based on the “puddle mode” and “lake model,” respectively. In the present experiment, high qP and qL in melatonin-treated wheat seedlings indicated that melatonin could effectively improved the fraction of open PSII reaction centers under osmotic stress. qN is mainly involved in carotenoid quenching of exactions, pH-dependent limitations in the chloroplast, and heat dissipation. Thus, the decline in qN under osmotic stress was due to the fact that there was no need for photoprotection in the presence of melatonin under osmotic stress. These results were further confirmed by the data from ETR and the capacity of O_2_ evolution. It has been shown that melatonin can protect photosystem and enhance photosynthetic capacity in vascular plants exposed to different environmental stresses (Yin et al., 2013). Recent studies showed that melatonin improved photosynthetic efficiency probably through regulating the CAND2/PMTR1-mediated signaling pathway (Bychkov et al., 2021; Yang et al., 2022), reducing chlorophyll degradation, and alleviating the damage to the integrity of thylakoid membrane *via* increasing the expression levels of electron-transport-related proteins under NO_2_ stress (Wang et al., 2022). In this study, exogenous melatonin application at 25 μM effectively improved the photosynthetic capacity of PSII under osmotic stress. These results are in line with the previous report of other plants under environmental stresses (Zhang et al., 2015; Chen et al., 2018a; Huang et al., 2019).

Non-photochemical quenching is the key photo-protection mechanism in photosynthesis, especially under environmental stresses. The excess light energy is harmlessly dissipated by NPQ in the form of heat. The high level of NPQ under stress conditions means high dissipation of excess light energy and low photochemical efficiency of photosynthesis (Graßes et al., 2002). The present study showed that osmotic stress significantly upregulated the level of NPQ, while melatonin application decreased NPQ level. The reason was probably because melatonin participated the regulation of xanthophyll cycle. Previous studies showed melatonin could enhance the size of xanthophyll pool and subsequently dissipated excess light energy *via* the stimulation of violaxanthin deepoxidase activity and enhancement of the deepoxidation of xanthophyll (Ding et al., 2017; Yan et al., 2021). These findings suggested that melatonin plays an important regulatory role in non-photochemical quenching under environmental stresses. State transition is another important protective mechanisms in response to environmental stresses through the excitation balance between PSI and PSII in plants (de Bianchi et al., 2011). In this study, the transition from state 1 to state 2 markedly declined under osmotic stress, but melatonin improved the level of state transition in wheat. The main reason was probably because that melatonin could effectively regulate the transiently reduced state of the free PQ pool under osmotic stress.

It has been known that the extent of damage to the photosynthetic apparatus can be effectively evaluated by OJIP analysis under environmental stresses (Zushi and Matsuzoe, 2017; Ayyaz et al., 2020). JIP-test parameters have also been applied in energy trapping, energy absorption, and electron transport in PSI and PSII in different areas of plant biology (Strasser et al., 2004). Recent studies demonstrated that melatonin application can mitigate the disruption of the photosynthetic electron transport on the receptor of PSII and thus alleviated the damage to oxygen evolving complex (OEC) of PSII under abiotic stresses through upregulating the expression levels of photosynthesis-related proteins (Zhang et al., 2021; Wang et al., 2022). In this paper, osmotic stress resulted in the significant decrease in the values of all JIP parameters related to the electron donor and acceptor sites of PSII, while melatonin obviously alleviated decline of JIP parameters, implying melatonin improved the amount of PSII reaction center (RC), enhanced electron transport flux, and finally protected OEC under osmotic stress. A previous report indicated that melatonin could maintain normal PSII function by protecting OEC under chromium stress (Ayyaz et al., 2020). The results were further verified by the data from O_2_ evolution rate of thylakoid membranes and also in line with several recent reports (Ayyaz et al., 2020; Zhang et al., 2021).

Melatonin might be associated with the post-transcriptional regulation of chloroplast gene expression and post-translational modification of chloroplast-related proteins (Yang et al., 2022). In addition, melatonin could improve the transcription of some photosynthetic genes and thus up-regulated their expression under different environmental stresses (Zhou et al., 2016; Chen et al., 2019; Alyammahi and Gururani, 2020; Zhang et al., 2021; Yang et al., 2022). Zhou et al. (2016) reported that melatonin influenced the translational step of D1 and thus counteracted the decline in the *de novo* synthesis of D1. A recent study demonstrated that melatonin treatment upregulated the expression of Lhcb5 and most Cab-related proteins under NO_2_ stress (Wang et al., 2022). In accordance with these findings, the western blot results showed that melatonin alleviated the decline in D1, Lhcb5, Lhcb6, PsbQ, and PsbS proteins under osmotic stress. D1 and PsbQ are mainly involved in the photoinhibition of PSII RC and the capacity of O_2_ evolution, respectively. Lhcb5, Lhcb6, and PsbS are related with the energy dissipation process. Therefore, our results suggested that exogenous melatonin could play the important regulatory roles in the photoinhibition of PSII, O_2_ evolution, and excitation energy dissipation under environmental stresses. These results were consistent with the data from photochemical efficiency of PSII, oxygen evolution rates, and NPQ.

It has been well known that reversible phosphorylation of thylakoid membrane proteins plays an important role in response to environmental stresses in land plants (Aro and Ohad, 2003; Vener, 2007; Chen et al., 2017a). Reversible phosphorylation of LHCII proteins is mainly associated with state transitions, thereby altering the energy balance between PSI and PSII (Pribil et al., 2010). Reversible phosphorylation of PSII RC proteins is involved in the repair cycle of PSII during environmental stresses (Tikkanen and Aro, 2012). Here, our results showed that osmotic stress induced the strong phosphorylation of PSII RC and LHCII proteins, while the application of melatonin obviously increased the dephosphorylation of PSII proteins in wheat exposed to osmotic stress. The reason might be due to the increase in the activities of protein phosphatases including PPH1 and PSII core phosphatase (PBCP) because melatonin can promote the activity of chloroplast enzymes (Yang et al., 2022). These findings indicated that melatonin could accelerate PSII repair cycle and maintain the balance of excitation energy between PSI and PSII through inducing the rapidly reversible phosphorylation of PSII proteins under environmental stresses.

## Conclusion

In summary, our results showed that osmotic stress resulted in the severe damage to PSII reaction centers and thus greatly reduce photosynthesis in wheat. However, melatonin could effectively provide the protective roles in photosynthetic machinery by increasing electron transfer efficiency of PSII, improving the dissipation of excess light energy, upregulating the levels of PSII-related proteins, and accelerating the reversible phosphorylation of thylakoid proteins under osmotic stress. Further study is needed in order to elucidate whether these photosynthesis-related proteins can directly bind melatonin to execute its function. Moreover, investigations are necessary to reveal the mechanism in which melatonin regulates the reversible phosphorylation of PSII proteins in the thylakoid under environmental stresses. Also, studying how melatonin work in the chloroplast could be useful to understand the protective role of melatonin in photosynthesis and future agricultural production.

## Data availability statement

The original contributions presented in the study are included in the article/**Supplementary material**; further inquiries can be directed to the corresponding authors.

## Ethics statement

The authors declare that the experiments were performed in compliance with the current laws of China.

## Author contributions

SL performed the experiments and analyzed the data. SL, X-FS, H-TM, and S-QL completed the first draft. Y-QS and Y-EC designed the experiment and edited the manuscript. J-YG worked together with SL to finish the experiment. MY, Z-WZ, SY, and H-YZ helped to analyze the data and revise the manuscript. All authors contributed to the article and approved the submitted version.

## Funding

This work was financially supported by Applied Basic Research Program of Sichuan Province (2020YJ0410), Science and Technology Innovation Program of Ya’an, Sichuan Science and Technology Program (2022YFH0068), and the National Natural Science Foundation of China (32102759).

## Conflict of interest

The authors declare that the research was conducted in the absence of any commercial or financial relationships that could be construed as a potential conflict of interest.

## Publisher’s note

All claims expressed in this article are solely those of the authors and do not necessarily represent those of their affiliated organizations, or those of the publisher, the editors and the reviewers. Any product that may be evaluated in this article, or claim that may be made by its manufacturer, is not guaranteed or endorsed by the publisher.
