# Supplementary material for: Exogenous melatonin improved photosynthetic efficiency of photosystem II by reversible phosphorylation of thylakoid proteins in wheat under osmotic stress
Source: Front Plant Sci. 2022 Aug 2;13:966181. doi: 10.3389/fpls.2022.966181 (PMC9380962; doi:10.3389/fpls.2022.966181)
Supplement: Supplementary file 1 [file Data_Sheet_1.doc]

**Supplementary data**

[**Exogenous melatonin improved photosynthetic efficiency of photosystem II by reversible phosphorylation of thylakoid proteins in wheat under osmotic stress**](https://www.frontiersin.org/articles/10.3389/fpls.2020.615942/full)

Shuai Lin^1^, Xiao-Fang Song^1^, Hao-Tian Mao^1^, Shuang-Qing Li^1^, Jie-Ying Gan^1^, Ming Yuan^1^, Zhong-Wei Zhang^2^, Shu Yuan^2^, Huai-Yu Zhang^1^, Yan-Qiu Su^3^,*, Yang-Er Chen^1^,*

*^1^ College of Life Sciences, Sichuan Agricultural University, Ya’an 625014, China,* *^2^ College of Resources, Sichuan Agricultural University, Chengdu 611130, China, ^3^ College of Life Science, Sichuan Normal University, Chengdu, China*

*Correspondence:

Yang-Er Chen

anty9826@163.com

Yan-Qiu Su

[snowdream215@163.com](mailto:snowdream215@163.com)

**Supplementary data contents**

Supplemental Figures 1-10

**Supplemental Figures 1-10**


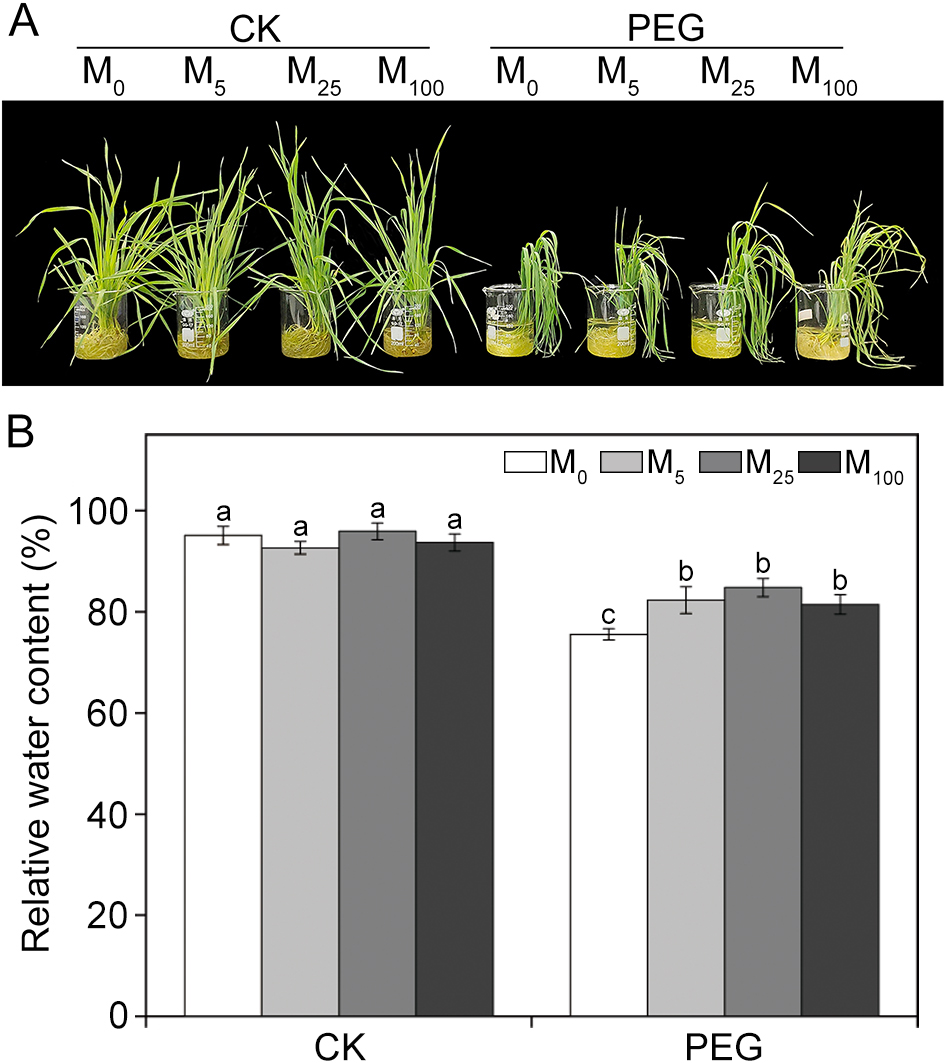


**FIGUR S1** Effects of melatonin on phenotype **(A)** and relative water content **(B)** in wheat seedlings under osmotic stress. Bars represent standard deviations from three independent biological replicates (*n* = 3). Different letters indicate significant differences (*P* < 0.05) according to Duncan’s multiplication range test. CK, non-stressed wheat plants; PEG, osmotic stress for 3 d; M_0_-M_100_ show 0, 5, 25, and 100 μM melatonin, respectively.


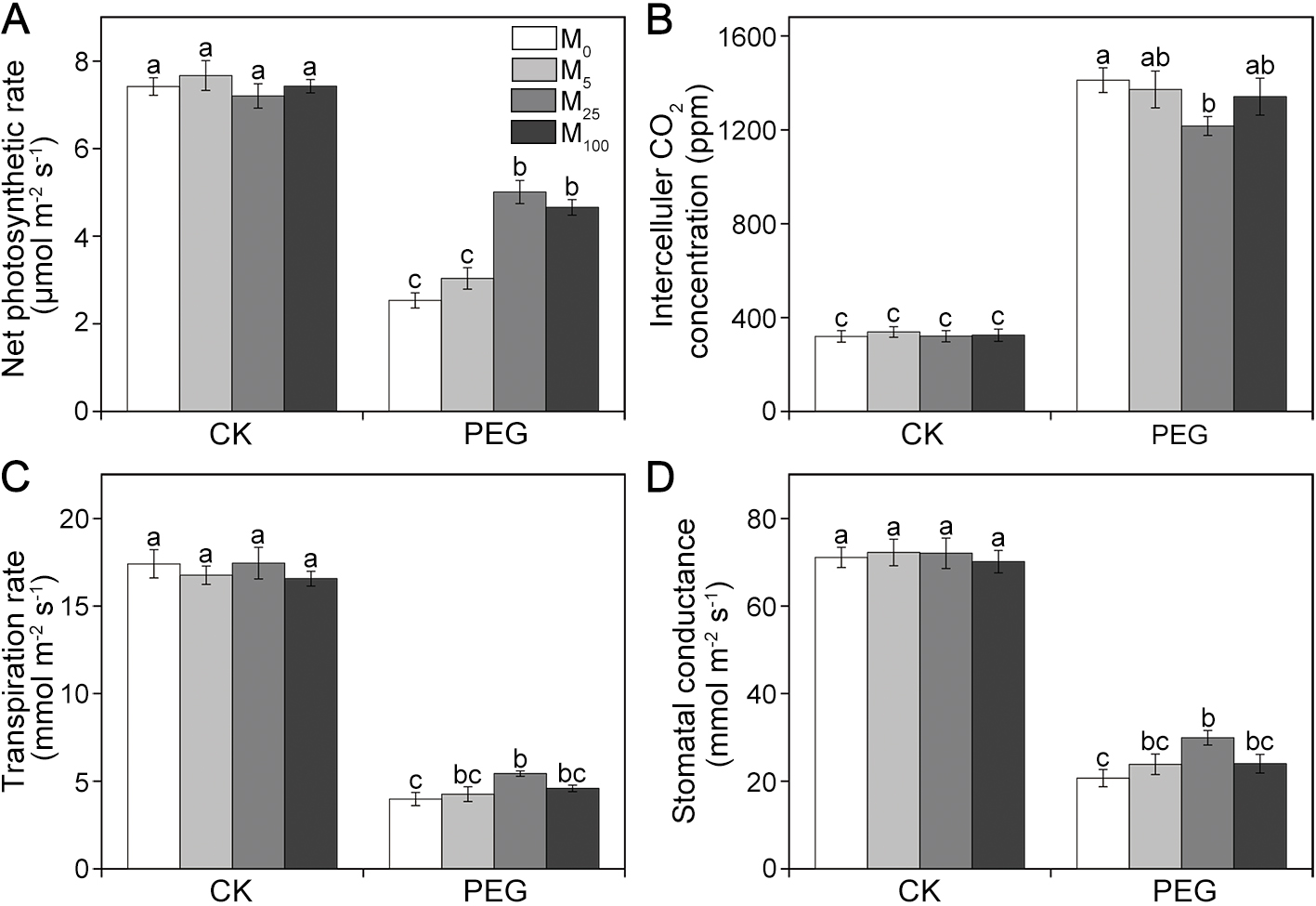


**FIGURE S2** Effects of melatonin on net photosynthetic rate **(A)**, intercellular CO_2_ concentration **(B)**, transpiration rate **(C)**, and stomatal conductance **(D)** in wheat seedlings under osmotic stress. Data is presented as mean ± SD for three replicates. Values followed by different letters are significantly different at *P* < 0.05 according to Duncan’s multiple range test. CK, non-stressed wheat plants; PEG, osmotic stress for 3 d; M_0_-M_100_ show 0, 5, 25, and 100 μM melatonin, respectively.


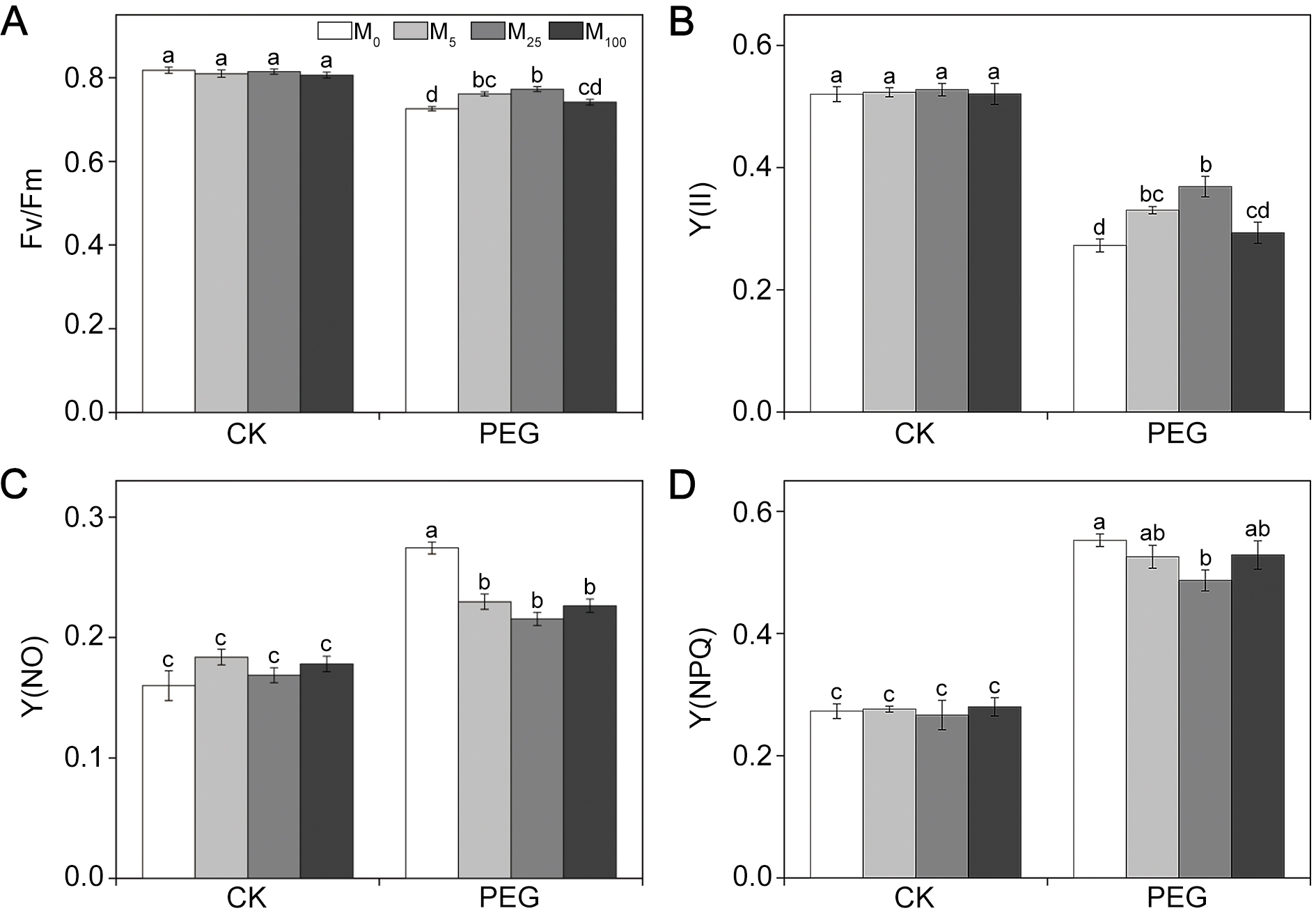


**FIGURE S3** Effects of melatonin on Fv/Fm **(A)**, Y(II) **(B)**, Y(NO) **(C)**, and Y(NPQ) **(D)** in wheat seedlings under osmotic stress. Data is presented as mean ± SD for three replicates. Values followed by different letters are significantly different at *P* < 0.05 according to Duncan’s multiple range test. CK, non-stressed wheat plants; PEG, osmotic stress for 3 d; M_0_-M_100_ show 0, 5, 25, and 100 μM melatonin, respectively.


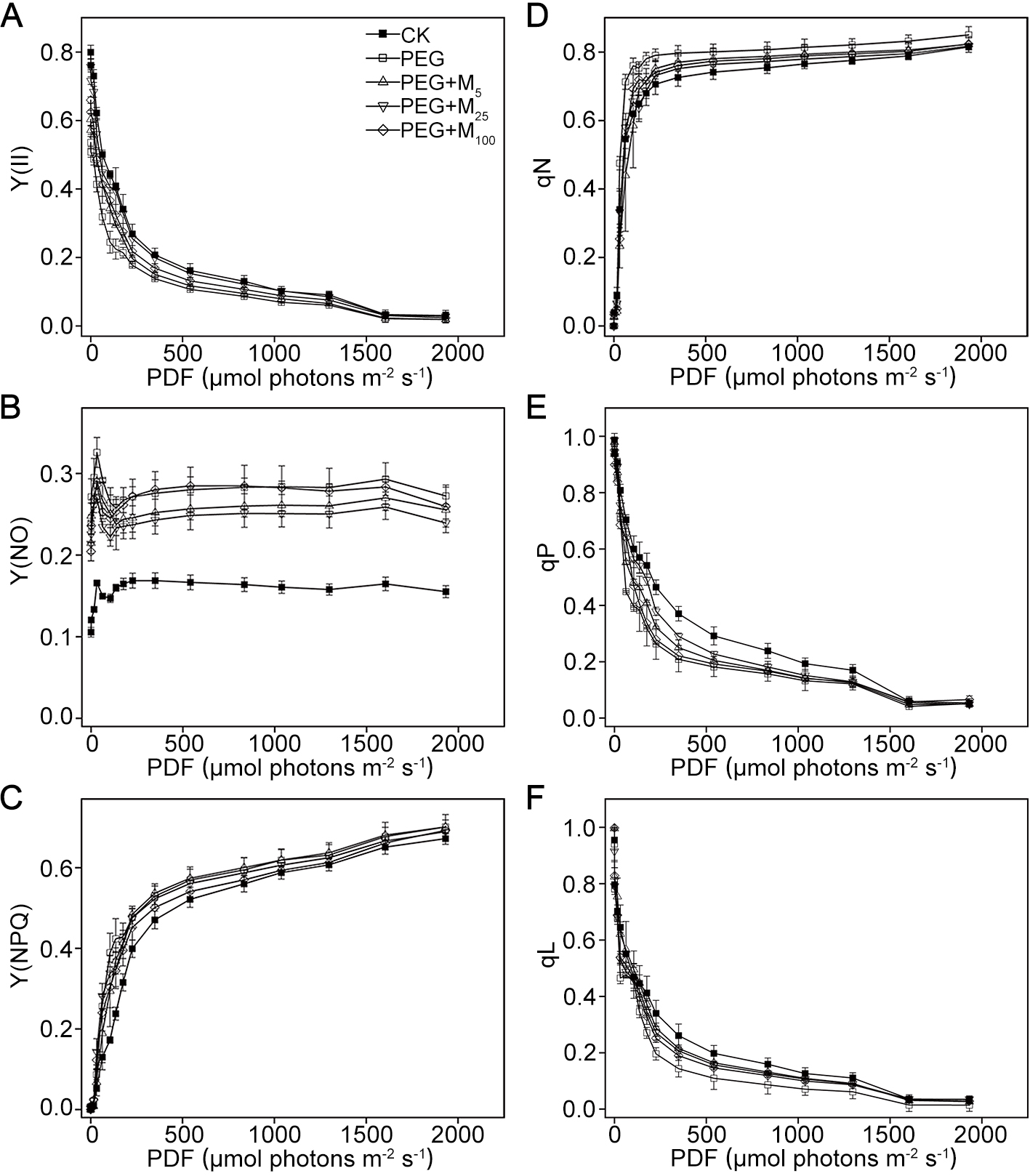


**FIGURE S4** Effects of melatonin on light response curves for Y(II) **(A)**, Y(NO) **(B)**, Y(NPQ) **(C)**, qN **(D)**, qP **(E)**, and qL **(F)** in wheat seedlings under osmotic stress. Bars represent standard deviations from three independent biological replicates (*n* = 3). CK, non-stressed wheat plants; PEG, osmotic stress for 3 d; M_5_-M_100_ show 5, 25, and 100 μM melatonin, respectively.


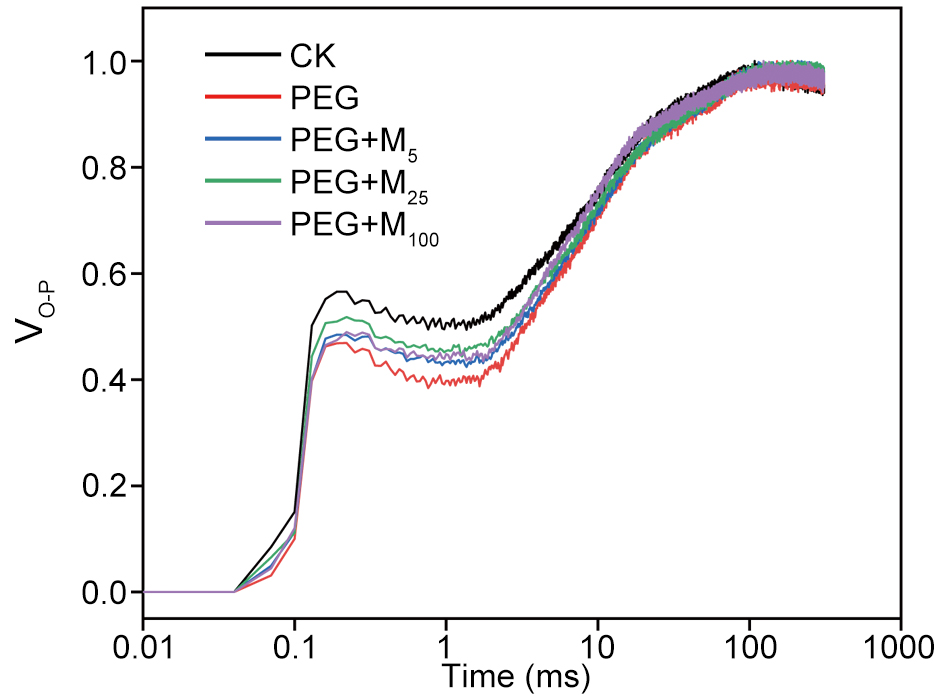


**FIGURE S5** Effects of melatonin on *V*_O-P_ curves in wheat seedlings under osmotic stress. The data in the figure are from three replicated experiments (*n* = 3). CK, non-stressed wheat plants; PEG, osmotic stress for 3 d; M_5_-M_100_ show 5, 25, and 100 μM melatonin, respectively.


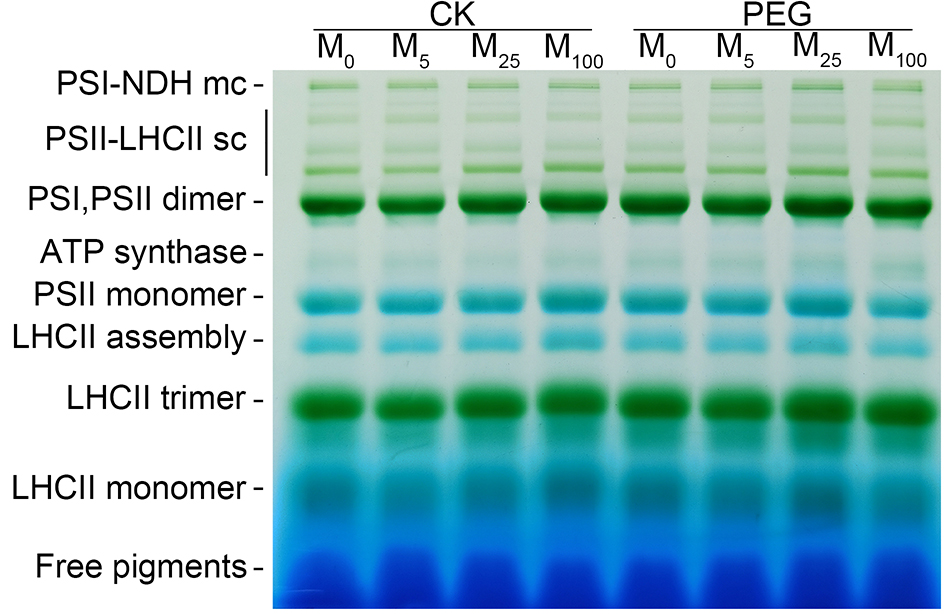


**FIGURE S6** Effects of melatonin on thylakoid membrane protein complexes in wheat seedlings under osmotic stress. CK, non-stressed wheat plants; PEG, osmotic stress for 3 d; M_0_-M_100_ show 0, 5, 25, and 100 μM melatonin, respectively.


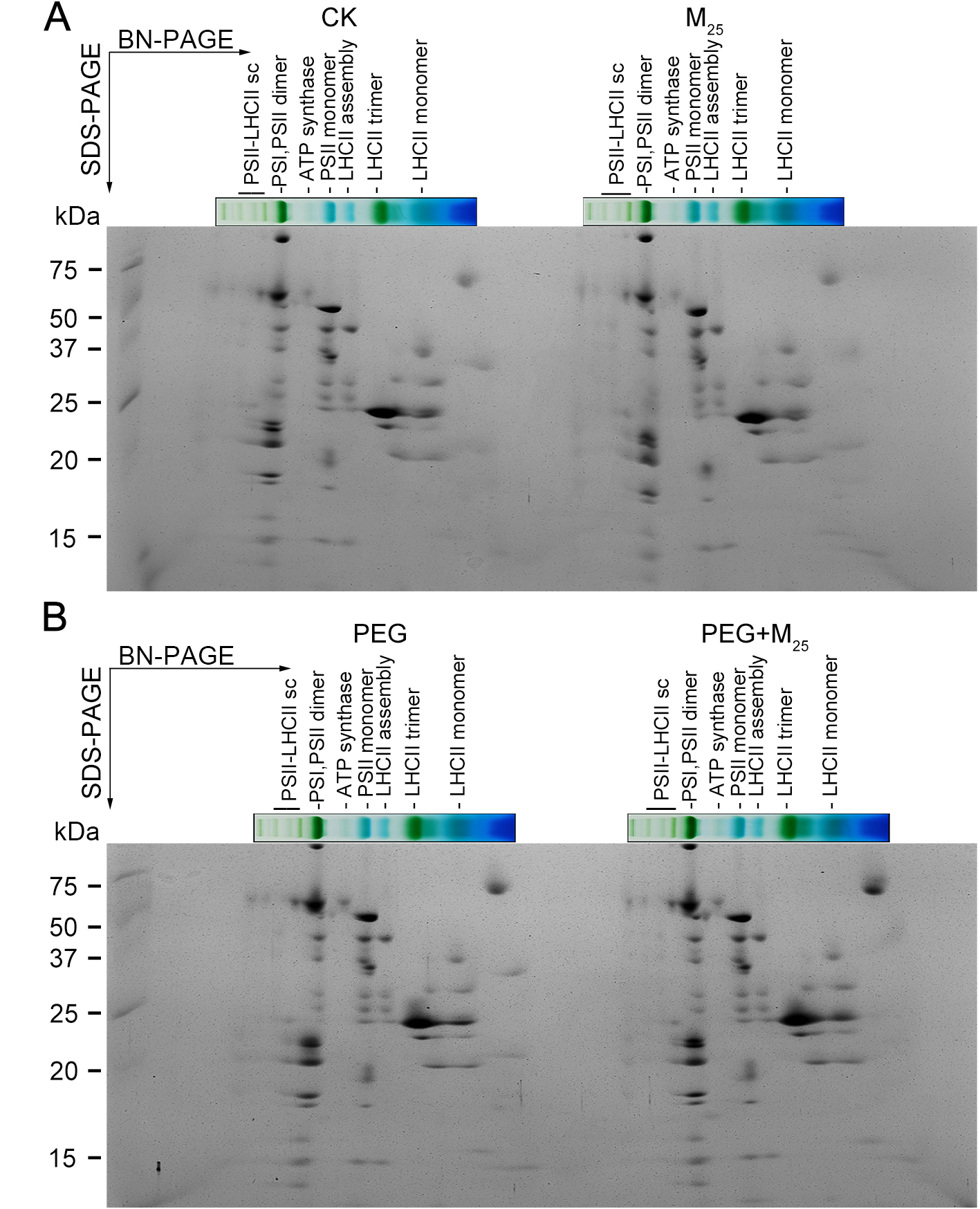


**FIGURE S7** Two-dimensional separation of thylakoid membrane protein complexes in wheat seedlings under osmotic stress. Thylakoid protein complexes separated in a BN-PAGE gel were subsequently separated in a second dimension by SDS-PAGE and Coomassie blue staining (CBS). CK, non-stressed wheat plants; PEG, osmotic stress for 3 d; M_25_, 25 μM melatonin.


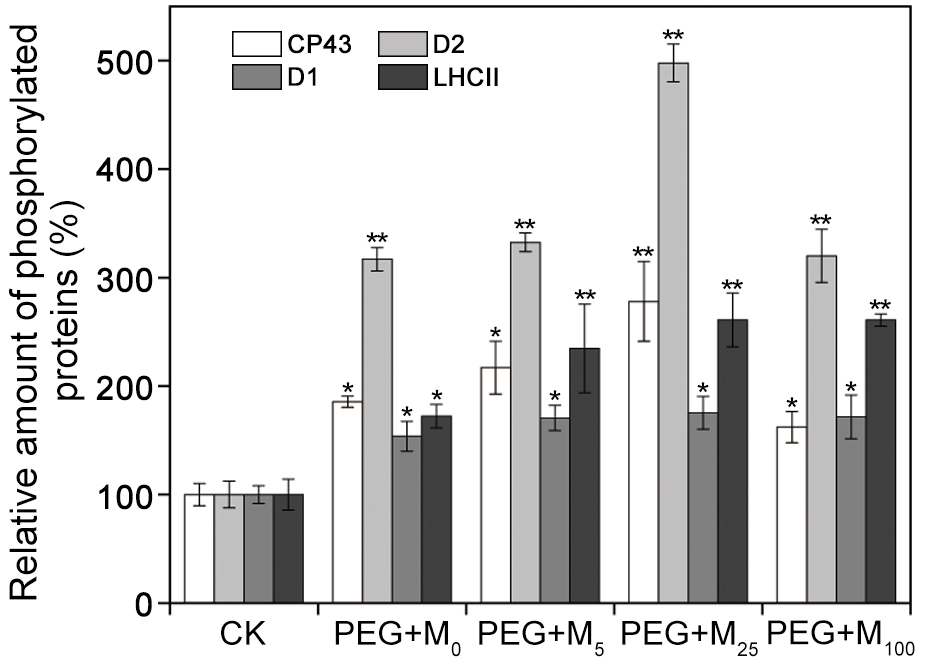


**FIGURE S8** Quantification of the immunoblots indicates the relative abundances of the phosphorylated proteins relative to the amount of control (100%). Values are the averages of 3 replicates ± SD. Asterisks indicate statistically significant differences at *P* < 0.05 level (Duncan’s multiple range test). CK, non-stressed wheat plants; PEG, osmotic stress for 3 d; M_0_-M_100_ show 0, 5, 25, and 100 μM melatonin, respectively.


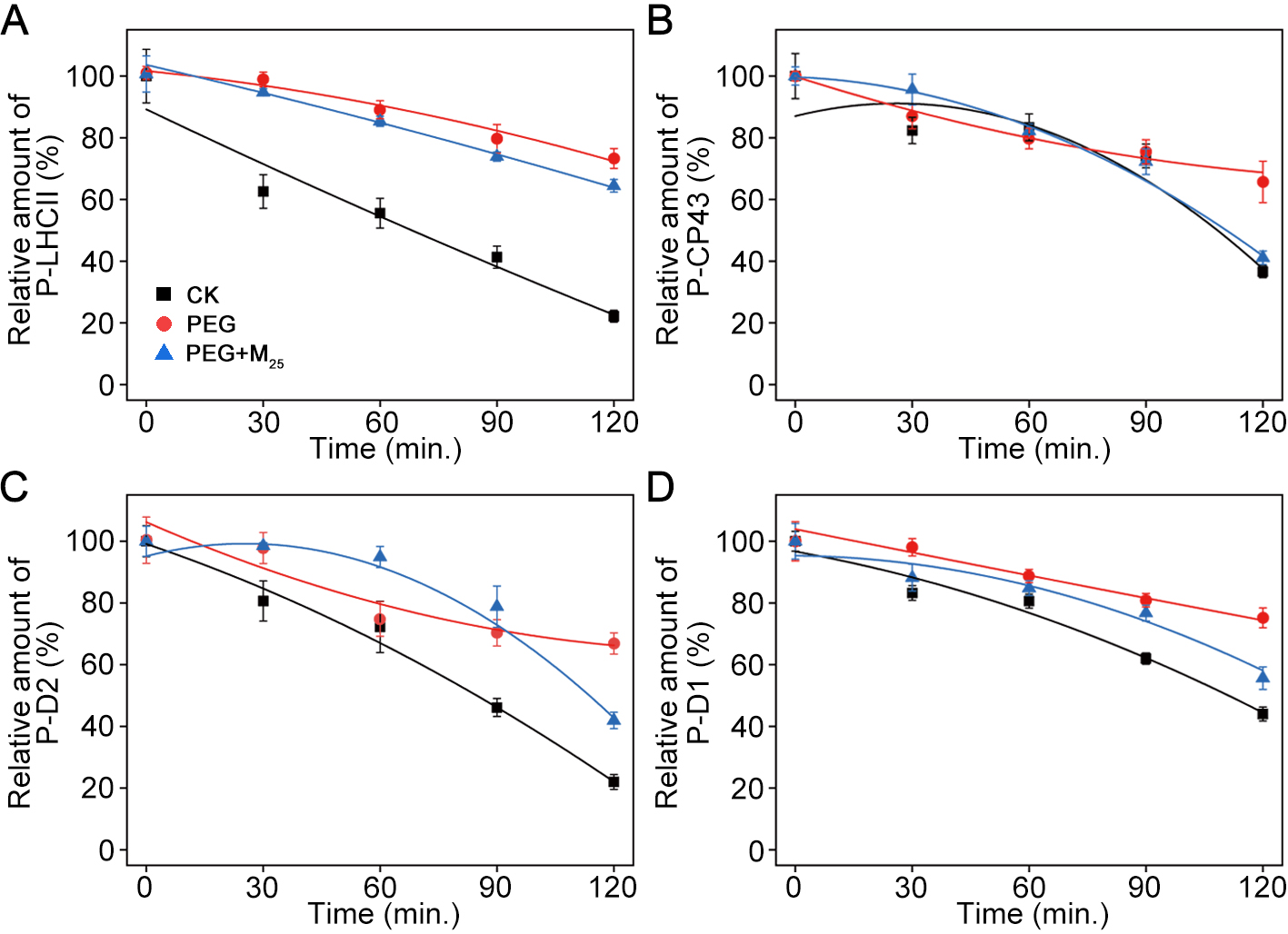


**FIGURE S9** Quantification of PSII protein dephosphorylation in wheat seedlings with 25 μM melatonin *in vivo* under osmotic stress. Results are presented relative to the amount of respective 0 min (100%). The line is a fit of all data to the descriptive hyperbolic equation y = ax^2^ + bx + c. CK, non-stressed wheat plants; PEG, osmotic stress for 3 d; M_25_, 25 μM melatonin.


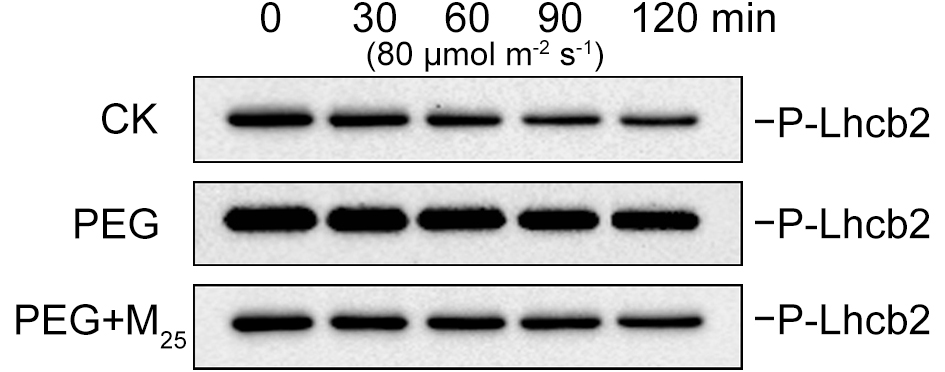


**FIGURE S10** Dephosphorylation of Lhcb2 from wheat seedlings with 25 μM melatonin *in vivo* under osmotic stress. CK, non-stressed wheat plants; PEG, osmotic stress for 3 d; M_25_, 25 μM melatonin.
